# Neonicotinoid pesticides disrupt gingival epithelial barrier function

**DOI:** 10.1016/j.toxrep.2026.102238

**Published:** 2026-03-16

**Authors:** Tsukasa Tamamori, Keita Tanigaki, Sasaki Naoko, Risako Matsumura, Shunsuke Yamaga, Akito Sakanaka, Hiromasa Tsujiguchi, Akinori Hara, Atsuo Amano, Michiya Matsusaki, Hiroyuki Nakamura, Hiroki Takeuchi, Masae Kuboniwa

**Affiliations:** a Department of Preventive Dentistry, Graduate School of Dentistry, The Osaka University, Suita-Osaka 565-0871, Japan; b Joint Research Laboratory (TOPPAN) for Advanced Cell Regulatory Chemistry, Graduate School of Engineering, The Osaka University, Suita‑Osaka 565‑0871, Japan; c Department of Preventive Dentistry, The Osaka University Dental Hospital, Suita-Osaka 565-0871, Japan; d Department of Hygiene and Public Health, Graduate School of Medical Sciences, The Kanazawa university, Kanazawa-Ishikawa 920-8640, Japan; e Environmental Stress Research Center, The Kanazawa university, Kanazawa-Ishikawa 920-8640, Japan; f Department of Applied Chemistry, Graduate School of Engineering, The Osaka University, Suita‑Osaka 565‑0871, Japan

**Keywords:** Neonicotinoid, Periodontitis, Pesticide, CXADR, Gingival epithelial cells, *Porphyromonas gingivalis*

## Abstract

Neonicotinoid pesticides are highly persistent in the environment, with detection in periodontal blood reported. Although an association with gingival inflammation has been shown, the mechanism related to periodontal disease remains unclear. Previous study found that coxsackievirus and adenovirus receptor (CXADR) is involved in gingival tissue epithelial barrier function, thus the effects of neonicotinoids on CXADR were examined. High performance liquid chromatography of salivary samples from 16 volunteers detected acetamiprid, clothianidin, imidacloprid, and thiamethoxam. Administration of neonicotinoids (Σneonicotinoids) resulted in loss of cell-surface CXADR, which was restored by bafilomycin A1, a lysosomal inhibitor. Using a three-dimensional tissue model of human gingival epithelial cells, Σneonicotinoids were found to increase permeability to lipopolysaccharide (LPS) and peptidoglycan (PGN), which was dependent on CXADR. It is thus suggested that neonicotinoids cause mislocalization of CXADR into lysosomes, leading to gingival barrier function disruption, which allows for bacterial virulence factors to penetrate subepithelial tissues.

## Highlights

• Neonicotinoid pesticides can be detected in saliva of Japanese residents with high performance liquid chromatography.
• Neonicotinoid disturbs intracellular transport of CXADR, a tight junction-related protein in gingival epithelial cells.
• Neonicotinoid pesticides increase permeability of gingival epithelial tissues to lipopolysaccharide and peptidoglycan.

## Graphical Abstract

**Figure:**
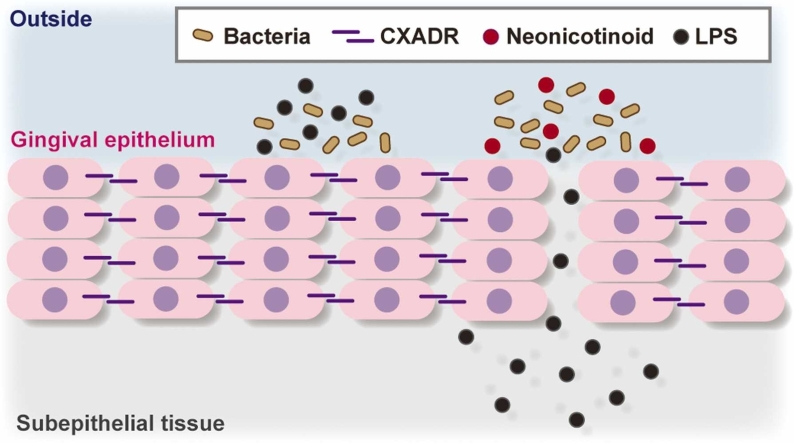

## 1 Introduction

Neonicotinoids, also known as pesticide residues, are widely used to provide insect protection for a variety of agricultural products, including cereals, oilseeds, fruits, and vegetables [1]. The first commercial neonicotinoid, imidacloprid (ICP), became available in the 1990s, followed by other compounds in subsequent years. These systemic agents were shown to consist of a diverse group of compounds and the term 'neonicotinoid' was proposed in 1993 [2]. Neonicotinoids, including acetamiprid (ATP), clothianidin (CTN), dinotefuran (DTR), ICP, nitenpyram (NTP), thiacloprid (TCP), and thiamethoxam (TMX), target insect nicotinic acetylcholine receptors (nAchRs), and subsequently cause overstimulation and paralysis in the nervous system of insects, leading to eventual death [1]. Since their introductions in the 1990s and early 2000s, neonicotinoids have spread rapidly, though concerns regarding environmental effects are growing, with effects on honey bees and other non-target organisms leading to concerns [3]. As a result, regulations and restrictions regarding use of neonicotinoids have been introduced in some countries and regions [4]. Because the half-lives of most neonicotinoids range from months to years, with repeated usage they persistently remain in the environment of aerobic soil and aquatic ecosystems for several years [5].

Following application to crops, neonicotinoids remain on the surface, while pesticide residues also invade their tissues [4] and are toxic to insects when eaten. Additionally, more than 90% of tea leaves sold for human consumption in Japan were found to contain two or more neonicotinoid compounds [6], while a study conducted in the United States found that 72% of fruits and 45% of vegetables examined were polluted with neonicotinoids [7]. Accordingly, there is rising concern regarding potential human exposure to neonicotinoids because of diet, as they have also been frequently detected in serum [8], urine [9], cerebrospinal fluid [10], and saliva and periodontal blood fluid [11].

In humans, CTN induces oxidative stress and DNA damage in bronchial epithelial cells [12], while ICP induces lysosome dysfunction and apoptosis in fibroblasts and astrocytes [13]. It has also been reported that periodontal disease is associated with exposure to neonicotinoids [14]. Periodontal disease is a chronic inflammatory disease initiated by bacterial infection and shown to have a multifactorial etiology [15], [16]. Since periodontal bacteria are known to directly interact with epithelial surfaces of the gingival sulcus, it is considered important to examine the effects of neonicotinoids on barrier function of gingival epithelium.

Coxsackievirus and adenovirus receptor (CXADR), a tight junction-related protein, has been shown to be expressed in gingival epithelial cells [17]. Additionally, it has been reported that CXADR contributes to barrier function against bacterial virulence factors of lipopolysaccharide (LPS) and peptidoglycan (PGN) [18]. Hence, tight junction-related proteins are considered to be an important target for research regarding residual pesticides in gingival epithelium. Because of ethical issues related to analysis of neonicotinoids in human tissues, gingival epithelial cells examined in the present study were cultured using a three-dimensional technique. The results clearly showed that neonicotinoids are environmental factors related to periodontal disease.

## 2 Materials and methods

### 2.1 Sample collection

All human subjects who participated provided informed consent to the study protocol, which was reviewed and approved by the ethics committee of The Osaka University Graduate School of Dentistry (R3-M1). All procedures were performed in accordance with the ethical guidelines for life sciences and medical research involving human subjects established by the Ministry of Health, Labour, and Welfare in Japan. Human saliva samples were collected in July 2024 during examinations conducted in Ishikawa prefecture in Japan. Volunteers without occupational exposure to neonicotinoids were selected and sixteen, including seven males and nine females, ranging in age from 43 to 78 years, were randomly selected to participate in this study. To avoid food resides in the mouth, drinking and eating from the night before the sampling day was prohibited. For sample collection, the participants were asked to sit in a dental unit chair in a relaxed position. Resting saliva (approximately 5 mL) was collected from each for 10 min into a 50-mL centrifuge tube (430052, Corning), which was placed on ice and all samples were immediately stored below −80°C until analysis.

### 2.2 Sample extraction

Prior to testing, the saliva samples were prepared as previously described [11], [19], [20], with minor modifications. Briefly, each sample was added to 0.025 ng/mL of acetonitrile containing six internal standards and vortexed. Centrifugation at 20,389 × *g* was performed for 10 min, then equal amounts of the supernatant were transferred to four new ultrafiltration filters. Following centrifugal filtration at 14,000 × *g* for 60 min, one of the four tubes was redissolved in 50% acetonitrile, with that solution then transferred to the remaining three tubes and concentrated in the same way. Finally, the solutions were transferred to polypropylene inserts and used as measurement samples.

### 2.3 Instrumental analysis

The human saliva samples were examined using liquid chromatography with tandem mass spectrometry (LC-MS/MS) by Infinity Lab (Yamagata, Japan; https://www.infinity-lab.jp/index.html), which provided highly sensitive quantitative analyses of 10 neonicotinoid-related substances, including ATP, CTN, DTR, ICP, NTP, TCP, TCP, and TMX. Isotope-labeled internal standards of ATP-d3 (39241, Sigma Aldrich), CTN-d3 (56816, Sigma Aldrich), DTR-d3 (99053207, HAYASHI), ICP-d4 (34170, Sigma Aldrich), TCP-d4 (30673, Sigma Aldrich), and TMX-d3 (38176, Sigma Aldrich), as well as the internal standard of NTP (CNLM-10545, CIL) were obtained from the indicated suppliers (Supplementary Figure 1). Detection of neonicotinoids was performed using an Agilent 6470B triple quadrupole mass spectrometer (Agilent 6470B Triple Quadrupole Mass Spectrometer; Agilent Technologies) equipped with an Agilent 1290 infinity LC system (Agilent 1290 infinity LC system; Agilent Technologies). Electrospray ionization in positive mode (ESI+) and multiple reaction monitoring (MRM) were used for identification and quantification of the target analytes, respectively. A Zorbax SB-C18 column (50 mm × 2.1 mm × 1.8 µm; Agilent) was employed to separate neonicotinoids, with an injection volume of 1 µL and the column temperature maintained at 45˚C.

A mixture of 0.1% formic acid and 5 mM ammonium formate in water (solvent A), and methanol (solvent B) were used as the mobile phase. Gradient elution was performed at a flow rate of 0.3 mL/minute as follows: 0–0.5 min, 5% solvent B; 0.5–6 min, 5–62%, 6–7 min, 62–100%, 7–9 min, and 100%, held for 4 min.

### 2.4 Cell cultures

Immortalized human gingival epithelial (IHGE) cells [21] were maintained in Humedia KG-2 (Kurabo). IHGE cells overexpressing CXADR were constructed, as previously described [18]. Three-dimensional cultures were then established as described in other studies [22], [23], with some modifications. Briefly, following trypsinization, IHGE cells were collected by centrifugation and incubated for three minutes with 0.2 mg/mL of fibronectin (Sigma-Aldrich) in a 0.1-mg/mL gelatin solution (Nacalai tesque). After three immersion steps, fibronectin/collagen nanofilms were coated onto the surfaces of individual cells. For tissue morphological analysis, a total of 2 × 10^6^ cells coated with fibronectin/collagen were seeded onto coverslips coated with vitronectin solution (A14700, Invitrogen) diluted 1/100 (v/v) in PBS in 24-well plates (Iwaki). After 24 h of incubation, the tissues were subjected to various experiments, then fixed and analyzed using a confocal microscope (TCS SP8; Leica Microsystems). For permeability experiments, 1 × 10^6^ cells coated with fibronectin/collagen were seeded into 24-well cell culture inserts (353096, Corning).

### 2.5 Antibodies and reagents

Antibodies and reagents used in this study are presented in Table S1. Neonicotinoids were conditioned in Humedia-KG2 medium at the following concentrations: 4.1 pg/mL of acetamiprid (DRE-C10013200, LGC Standards), 22.3 pg/mL of clothianidin (C3746, Tokyo Chemical Industry), 16.7 pg/mL of imidacloprid (I1145, Tokyo Chemical Industry), and 8.5 pg/mL of thiamethoxam (T3811, Tokyo Chemical Industry). In the control condition, IHGE cells or tissues were treated with the same volume of distilled water used to dissolve the neonicotinoid, without the neonicotinoid itself. Those concentrations were determined based on the average number of positive cases detected at each concentration (Table 1). The values shown were rounded to the second decimal place.

**Table 1:** Neonicotinoid concentrations in human saliva collected from subjects in Ishikawa prefecture in Japan.

Sample No.	Neonicotinoid concentration (ng/mL)						
	ATP	CTN	DTR	ICP	NTP	TCP	TMX
1	0.005	0.025	*N.D.	0.018	*N.D.	*N.D.	0.008
2	*N.D.	*N.D.	*N.D.	*N.D.	*N.D.	*N.D.	0.003
3	*N.D.	*N.D.	*N.D.	*N.D.	*N.D.	*N.D.	*N.D.
4	*N.D.	0.026	*N.D.	*N.D.	*N.D.	*N.D.	0.027
5	0.003	*N.D.	*N.D.	0.030	*N.D.	*N.D.	0.006
6	*N.D.	*N.D.	*N.D.	*N.D.	*N.D.	*N.D.	0.004
7	0.007	*N.D.	*N.D.	0.017	*N.D.	*N.D.	0.006
8	0.004	*N.D.	*N.D.	*N.D.	*N.D.	*N.D.	0.009
9	*N.D.	*N.D.	*N.D.	*N.D.	*N.D.	*N.D.	*N.D.
10	0.003	*N.D.	*N.D.	0.025	*N.D.	*N.D.	0.020
11	0.040	*N.D.	*N.D.	0.008	*N.D.	*N.D.	0.006
12	0.002	0.016	*N.D.	0.009	*N.D.	*N.D.	0.006
13	0.004	*N.D.	*N.D.	0.010	*N.D.	*N.D.	0.005
14	0.005	*N.D.	*N.D.	*N.D.	*N.D.	*N.D.	0.006
15	*N.D.	*N.D.	*N.D.	*N.D.	*N.D.	*N.D.	*N.D.
16	*N.D.	*N.D.	*N.D.	*N.D.	*N.D.	*N.D.	0.004

Abbreviations: ATP, acetamiprid; CTN, clothianidin; DTR, dinotefuran; ICP, imidacloprid; NTP, nitenpyram; TCP, thiacloprid; TMX, thiamethoxam. The salivary concentration (ng/mL) was measured with LC-MS/MS. *N.D., not detected.

### 2.6 Immunoblotting and immunocytochemistry

Immunoblotting and immunocytochemistry were performed as previously described [22]. Immunoreactive bands were detected using Pierce ELC Western Blotting Substrate (Thermo Scientific) and ChemiDoc XRS (Bio Rad), then images were acquired with the Quantify One software package (Bio-Rad). Confocal microscopic images were acquired with a confocal laser microscope (TCS SP8; Leica Microsystems) equipped with a 64 × oil-immersion object lens with a numerical aperture of 1.4, then analyzed using the Application Suite X software package (Leica Microsystems).

### 2.7 Quantitative real-time PCR (qRT-PCR)

Quantitative real-time PCR was performed as previously described [22]. Total RNA was extracted from IHGE cells using an RNeasy Micro kit (Qiagen). Complementary DNA was synthesized using ReverTra Ace qPCR RT Master Mix (Toyobo). Real-time PCR was performed using a Rotor Gene Q (Qiagen) with THUNDERBIRD SYBR qPCR Mix (Toyobo). The primer sequences are shown in Table S2. The amplicon level in each sample was normalized based on the corresponding level of *β*-*ACTIN* mRNA content using the 2^−ΔΔCt^ method.

### 2.8 Epithelial barrier functional assay

FITC-tracers were prepared as previously described [22]. To assess barrier function, *in vitro* epithelial permeability assays were performed with 24-well culture inserts (353096; Corning) using a previously reported method [22]. *P. gingivalis* LPS (14F18-MM, InvivoGen) and *S. aureus* PGN (77140, Sigma-Aldrich) were labeled with FITC using a Fluorescein Labeling Kit-NH2 (LK-01, Dojindo). Prior to the assays, FITC-labeled LPS was incubated with 10 mM citrate (Wako) and 0.05% (v/v) Tween-20 (Calbiochem) for 45 min at 37°C, as previously described [24], while FITC-labeled PGN was incubated with 0.5 mg/mL lysozyme (Nacalai Tesque) for 45 min at 37°C to obtain a suspension. Twenty microliters of FITC-LPS or FITC-PGN at 0.1 mg/mL was then added to the upper compartment of the culture insert. Following incubation, medium was collected from the lower compartment and fluorescence intensity determined using a 1420 ARVO X (PerkinElmer). Data obtained were analyzed using the WorkOut Plus software package (PerkinElmer).

### 2.9 Statistical analysis

P values were determined using a *t* test with the Excel software package (Microsoft), with p < 0.05 considered to indicate significance.

## 3 Results

### 3.1 Detection of neonicotinoids in human saliva

Detection frequency and concentrations of neonicotinoids in saliva samples collected from subjects in Ishikawa prefecture in Japan are presented in Table 1. TMX was most frequently detected (81% of the samples), followed by ATP, ICP, and CTN (56%, 44%, 19% respectively), whereas DTR, NTP, and TCP were not detected. The average concentrations of neonicotinoids in the positive samples were 4.1 pg/mL for ATP, 22.3 pg/mL for CTN, 16.7 pg/mL for ICP, and 8.5 pg/mL for TMX, while their median concentrations were 4 pg/mL, 25 pg/mL, 17 pg/mL, and 6 pg/mL, respectively. In these samples, CTN had the highest average and median concentration levels.

### 3.2 Effects of neonicotinoid on barrier function of gingival epithelial cell layers

**Fig. 1:**
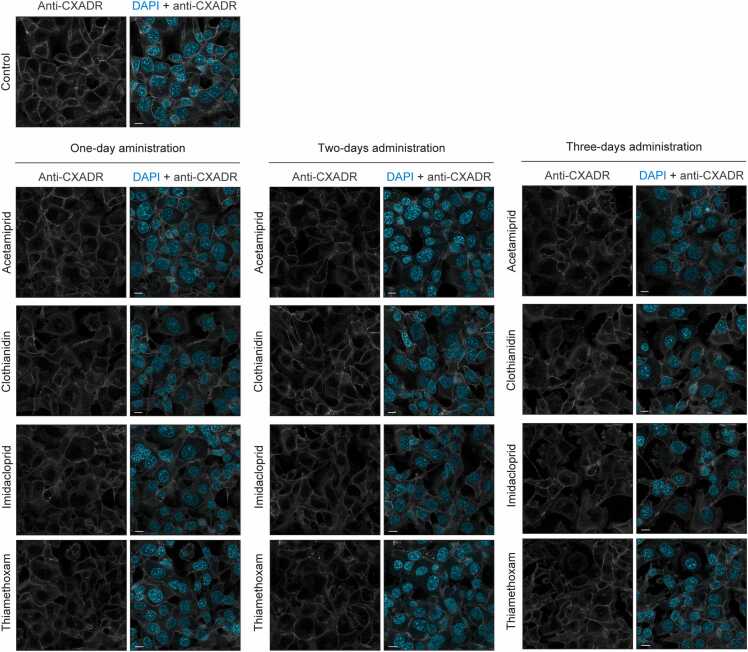
CXADR localization in IHGE cells disturbed by neonicotinoids. IHGE cells were separately exposed to each neonicotinoid (ATP [4.1 pg/mL], CTN [22.3 pg/mL], ICP [16.7 pg/mL], TMX [8.5 pg/mL]) for the indicated time periods. The cells were then fixed, stained with DAPI (cyan) and rabbit monoclonal anti-CXADR (gray: Alexa Fluor 555), and analyzed using confocal microscopy. Scale bars, 10 μm.

The effect of neonicotinoid on CXADR distribution in gingival epithelial cells was examined, based on the average concentrations in positive samples (Table 1). CXADR was found located on the surface of IHGE cells, while localization was lost in a time-dependent manner in cells treated with eATP, CTN, ICP, or TMX (Fig. 1). These results suggest that neonicotinoids decrease cell-surface CXADR on human gingival epithelial cells.

### 3.3 CXADR mislocalization in lysosomes following neonicotinoid administration

**Fig. 2:**
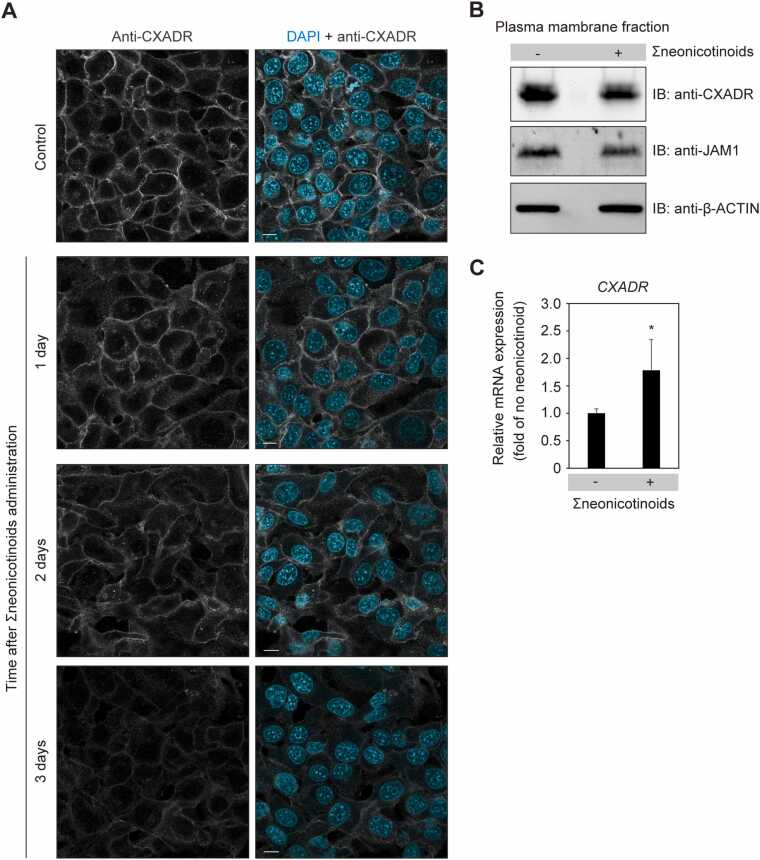
CXADR localization in IHGE cells disturbed by Σneonicotinoids. (A) IHGE cells were exposed to Σneonicotinoids (ATP [4.1 pg/mL], CTN [22.3 pg/mL], ICP [16.7 pg/mL], TMX [8.5 pg/mL]) for the indicated time periods. The cells were then fixed, stained with DAPI (cyan) and rabbit monoclonal anti-CXADR (gray: Alexa Fluor 555), and analyzed using confocal microscopy. Scale bars, 10 μm. (B) IHGE cells were exposed to Σneonicotinoids for three days, then analyzed using immunoblotting with the indicated antibodies. β-ACTIN was used as a loading control. IB, immunoblot. Images of full-length blots are shown in Supplementary Figure 4. (C) Relative levels of *CXADR* mRNA expression in IHGE cells exposed to Σneonicotinoids. Cells were treated for three days, then samples were subjected to qRT-PCR assays. Results are expressed as fold change relative to no administration, with five technical replicates performed. The significance of differences was evaluated using a two-tailed *t* test. Data shown are representative of three biological replicates.

To examine the combined effects of ATP, CTN, ICP, and TMX (Σneonicotinoids) on CXADR, localization and level of these proteins were analyzed using confocal microscopy and immunoblotting. The findings confirmed that CXADR cell surface localization was lost and protein level reduced (Fig. 2A, 2B) by exposure to Σneonicotinoids at an average level detected in saliva (Table 1). It has been shown that junctional adhesion molecule 1 (JAM1) is also involved in the barrier function of gingival epithelial tissues [22], thus the protein level of JAM1 in plasma membrane fractions was also analyzed. As shown in Fig. 2B, a slight decrease of JAM1 by Σneonicotinoids was observed, though less as compared to CXADR, showing the latter as their primary target. To assess contributions of Σneonicotinoids toward *CXADR* gene expression, quantitative real-time PCR was performed. Σneonicotinoids did not decrease *CXADR*, indicating that a decreased level of CXADR protein by Σneonicotinoids is not due to downregulation of gene expression (Fig. 2C).

### 3.4 Mislocalization of CXADR in lysosomes by neonicotinoids

**Fig. 3:**
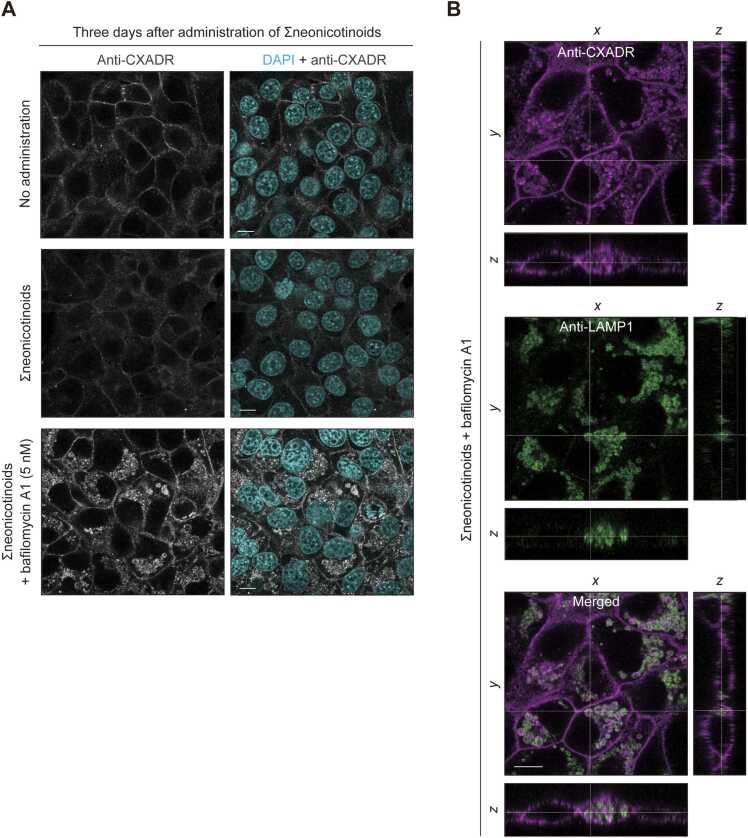
Lysosomal localization of CXADR in IHGE cells exposed to Σneonicotinoids. (A) IHGE cells were exposed to Σneonicotinoids with bafilomycin A1 (5 nM) for three days. The cells were then fixed, stained with DAPI (cyan) or rabbit monoclonal anti-CXADR (gray: Alexa Fluor 555), and analyzed using confocal microscopy. (B) IHGE cells were exposed to Σneonicotinoids with bafilomycin A1 (5 nM) for three days. The cells were then fixed, stained with rabbit monoclonal anti-CXADR (magenta: Alexa Fluor 555) or mouse monoclonal anti-LAMP1 (green: Alexa Fluor 488), and analyzed using confocal microscopy. Scale bars, 10 μm.

To assess the contributions of Σneonicotinoids to lysosomal degradation of CXADR, IHGE cells were treated with Σneonicotinoids in the presence or absence of bafilomycin A1, an inhibitor of lysosomal acidification [25], then involvement of lysosomal degradation of CXADR was analyzed using confocal microscopy findings. Treatment with bafilomycin A1 resulted in successful restoration of CXADR protein level (Fig. 3A). Additionally, abundant co-localization of CXADR and LAMP1, a lysosomal marker, was observed in Σneonicotinoids-treated cells in the presence of bafilomycin A1 (Fig. 3B). These results suggest that Σneonicotinoids cause mislocalization of CXADR in gingival epithelial cell lysosomes.

### 3.5 Induction of LPS and PGN penetration by Σneonicotinoids dependent on CXADR

**Fig. 4:**
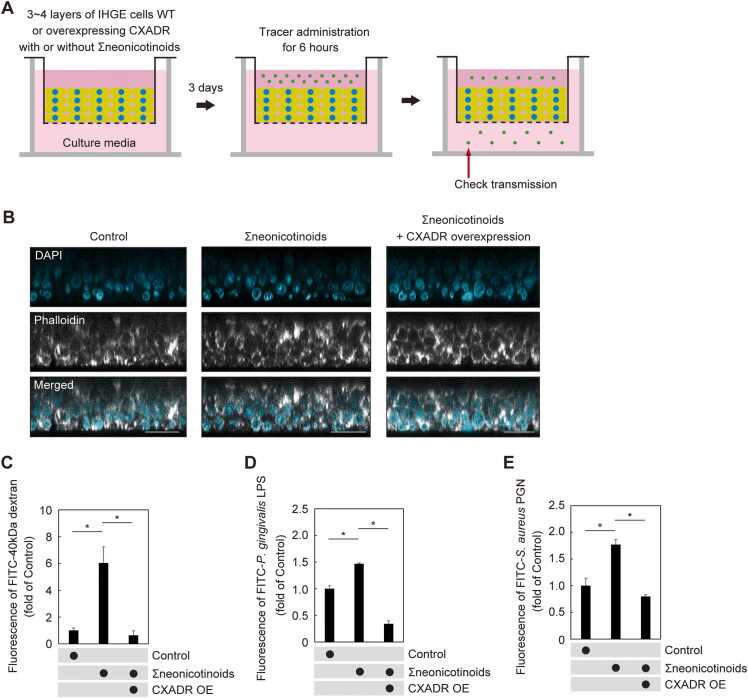
Epithelial barrier dysfunction of gingival epithelial tissues mediated by Σneonicotinoids. (A) Schematic image of culture-insert system. Gingival epithelial tissues in culture inserts were treated with Σneonicotinoids. Following two days of incubation, FITC-labeled tracers were added to the upper compartment. Following six hours of incubation, transmission of tracers from upper to lower compartment was analyzed by spectrometry. (B) Confocal microscopic cross-sectional images of 3D-tissue model of WT IHGE cells or those overexpressing CXADR. Gingival epithelial tissues on coverslips were exposed to Σneonicotinoids for two days, then fixed, stained with DAPI (cyan) or Alexa Fluor 633 (gray), and analyzed using confocal microscopy. Scale bars, 30 μm. (C-E) Permeability to FITC-40 kDa dextran (C), *P. gingivalis* LPS (D) or *S. aureus* PGN (E) in gingival epithelial tissues treated with the neonicotinoids. Results are expressed as fold change relative to the control (WT tissue without Σneonicotinoids) and presented as the mean ± SD of seven technical replicates. *p < 0.05, two-tailed *t* test (closed-testing procedure). Data shown are representative of two biological replicates.

To clarify the contribution of Σneonicotinoids to permeability of gingival epithelial tissues, a three dimensional-tissue model was generated using a cell accumulation technique [22], [23] (Fig. 4A). To confirm involvement of CXADR in the effects of neonicotinoids on barrier function, based on the level of reduction shown in Fig. 2A, IHGE cells overexpressing CXADR were employed for permeability assays [18]. CXADR overexpression enabled confirmation of effective compensation of cell-surface CXADR in Σneonicotinoids-treated cells (Supplementary Fig. 3 A and 3B). As shown in Fig. 4B, multilayers of wild-type (WT) gingival epithelial cells or those with CXADR overexpression were confirmed using the tissue model with or without neonicotinoids. At 48 h after Σneonicotinoids administration, FITC-40 kDa dextran, *P. gingivalis* LPS, or *Streptococcus aureus* PGN were added to the tissues and permeability assays conducted. Six hours after tracer administration, permeability to each of those was significantly increased by Σneonicotinoids (Fig. 4C-E), while permeation was canceled by CXADR overexpression.

These results suggest that neonicotinoids can cause harm to the barrier function of human gingival epithelium. Nevertheless, other environmental issues related to protection of periodontal tissue health must be taken into consideration.

## 4 Discussion

This is the first known study to reveal the molecular basis of pathogen-associated molecular pattern penetration through human stratified squamous epithelium mediated by a pesticide, supporting the hypothesis that neonicotinoids are a potential risk factor for periodontal health. The findings confirmed that people in Japan have been exposed to neonicotinoids at levels that can be detected in saliva. This study aims to demonstrate that pesticides migrate into human saliva and to identify a concentration range, which can be a basis for exposure levels in gingival cellular experiments. Pesticide concentrations in saliva are influenced by several factors, such as dietary intake, collection timing, and saliva matrix. Notably, dietary restriction before saliva sampling is needed to analyze the transition of pesticide from body circulation into saliva. Additionally, LC/MS analysis involves the issues of matrix effects and pre-treatment, high-throughput measurement is difficult at this stage. Consequently, the study was conducted with 16 subjects as the feasible sample size under our rigid experimental conditions. To determine exposure distribution within a population, large-scale analysis will be expected.

Saliva and gingival crevicular fluid, two major biological fluids around gingival epithelium, differ in their origin, composition, and biological characteristics. Saliva mainly reflects salivary gland secretion and systemic circulation, while gingival crevicular fluid derives from local vasculature and is dependent on periodontal inflammatory status. To clarify the primary effects of the pesticide on the gingival epithelial barrier, especially considering the onset of periodontitis, we employed saliva as stable exposure indicator.

Pesticide residues have some unique characteristics. As compared to periodontal bacteria, tobacco, and diabetes, all human generations have been widely exposed to neonicotinoids. For example, multiple neonicotinoids have been detected in cerebro-spinal fluid, plasma, and urine samples obtained from children [26], [10]. Additionally, even when the levels of pesticide residues in individual food types are within the agreed standard, individuals can become overexposed when such foods are consumed in combination. Also, it has been shown that neonicotinoids are metabolized by hepatic enzymes and aldehyde oxidases [27], [28], and those metabolites become distributed throughout the human body. Nevertheless, even though metabolized as well as parent neonicotinoids can be detected in periodontal blood fluid [11], details regarding the effects of such metabolites on the host remain largely unknown. Neonicotinoids are also widely used in materials employed for building, gardening, termite control, household insecticides, and pet flea control, and aerially sprayed in some situations. Therefore, it is important to evaluate the health effects of all forms of neonicotinoids as part of the exposome [29], which is the totality of complex environmental factors experienced throughout life.

Previous studies have found that vertebrae receptors are weakly sensitive to neonicotinoids including CTN and ICP [30], [31], [32], with nAChRs known to be targeted. When used as insecticides, neonicotinoids competitively modulate insect nAChRs. In contrast to those found in insects, mammalian nAChRs are expressed not only in the nervous system, but also in a wide range of tissues in the immune system and epithelia, as well as reproductive organs such as the placenta and ovaries [33]. nAChRs are classified into skeletal muscle-type (muscle-type) and neuronal-type (ganglion-type) based on various subunit genes (*CHRNA1*-*CHRNA10*, *CHRNB1*-*CHRNB4*, *CHRND*, *CHRNE*, *CHRNG*), making it difficult to assess the effects of individual receptor types on tissue phenotypes using a gene knockdown or knockout approach. Therefore, it is considered that use of highly selective inhibitors or genetic engineering techniques with fewer off-target effects will enable identification of neonicotinoid target molecules in host cells. Additionally, a previous study presented findings indicating intracellular uptake of acetamiprid via a sodium-dependent transporter and an ATP-dependent efflux transporter [34], suggesting both direct and indirect associations of neonicotinoids with cytosolic proteins.

The discrepancy observed between increased mRNA expression and reduced protein levels of CXADR can be explained, at least in part, by enhanced lysosomal degradation following pesticide exposure (Fig. 3). Environmental stressors has been shown to induce the disassembly of tight junction complexes and trigger endocytic removal of junctional proteins from the plasma membrane, followed by lysosomal targeting and degradation [35], [36]. Such post-translational regulation can result in a loss of junctional proteins despite compensatory upregulation at the transcriptional level. In this context, the increased expression of tight junction–related genes observed in our study may represent an incomplete or unsuccessful repair response to barrier disruption, while the concomitant lysosomal degradation prevents effective restoration of epithelial integrity. This post-translational mechanism may be relevant under pesticide exposure at a chronic low level, where sustained cellular stress impairs proper junctional reassemble and compromises barrier homeostasis.

The CXADR intracellular domain (ICD) contains the S-acylation sites cysteine 259 and 260 for palmitoylation, which is crucial for both internalization and recycling of membrane-bound CXADR [37]. Additionally, the ICD of CXADR interacts with PDZ domain-containing proteins, including ZO1 [38], MAGI1 [39], PICK1 and PSD95 [40], MUPP1 [41], and LNX [42], via the PDZ-binding motif [40]. It is thus considered that dysfunction of CXADR palmitoylation and PDZ domain-binding proteins may be involved in mislocalization of CXADR to lysosomes by neonicotinoids. On the other hand, JAM1 undergoes phosphorylation [43] or glycosylation [44], modifications involved in the barrier function of epithelial cells. Therefore, abnormal post-translational modification of JAM1 may be a key point to better understand the effects of neonicotinoids on host cells.

Neonicotinoids have been shown to have relationships with various conditions, including cancer [45], child development [46], neonatal birth size [47], and infertility [48]. Additionally, a broad range of agrochemicals, including arsenic-based herbicides [49] and acetochlor [50], [51], has been studied. Hence, the present findings will lead to more detailed molecular biological research to analyze the effects of not only neonicotinoids but also other agrochemicals on diseases. Additionally, for ethical considerations in intervention, the cell accumulation technique used in the present study would be helpful to analyze the effects of pesticides on tissues at genetic and protein levels.

## CRediT authorship contribution statement

**Risako Matsumura:** Resources, Investigation, Formal analysis. **Shunsuke Yamaga:** Resources. **Akito Sakanaka:** Resources. **Hiromasa Tsujiguchi:** Resources. **Masae Kuboniwa:** Writing – review & editing, Resources, Project administration. **Keita Tanigaki:** Writing – original draft, Resources, Investigation, Formal analysis. **Sasaki Naoko:** Resources. **Tsukasa Tamamori:** Writing – original draft, Resources, Investigation, Formal analysis. **Akinori Hara:** Resources. **Atsuo Amano:** Writing – review & editing, Supervision, Conceptualization. **Michiya Matsusaki:** Resources. **Hiroyuki Nakamura:** Resources. **Hiroki Takeuchi:** Writing – review & editing, Writing – original draft, Visualization, Validation, Supervision, Software, Resources, Project administration, Methodology, Investigation, Funding acquisition, Formal analysis, Data curation, Conceptualization.

## Declaration of Competing Interest

The authors declare the following financial interests/personal relationships which may be considered as potential competing interests: Hiroki Takeuchi reports financial support was provided by The Japan Society for the Promotion of Science. Atsuo Amano reports financial support was provided by The Japan Society for the Promotion of Science. If there are other authors, they declare that they have no known competing financial interests or personal relationships that could have appeared to influence the work reported in this paper.

## Data Availability

Data will be made available on request.
