# Supplementary material for: Neonicotinoid pesticides disrupt gingival epithelial barrier function
Source: Toxicol Rep. 2026 Mar 16;16:102238. doi: 10.1016/j.toxrep.2026.102238 (PMC13015243; doi:10.1016/j.toxrep.2026.102238)
Supplement: Supplementary file 1 — Supplementary material [file mmc1.docx]

**Supplementary information**

**Table S1. Antibodies and reagents.**

**Table S2. Primers for qRT-PCR.**

**Figure S1. Chemical structures of neonicotinoids used for LC-MS/MS.**

**Figure S2. Chemical structures of neonicotinoids used for cellular experiments.**

**Figure S3. Confocal microscopic images of Σneonicotinoid-treated IHGE cells overexpressing *CXADR*.**

**(A)** WT IHGE cells or those expressing HA-inserted CXADR were exposed to Σneonicotinoids for three days. The cells were then fixed, stained with DAPI (cyan) or rabbit monoclonal anti-CXADR (gray: Alexa Fluor 555), and analyzed using confocal microscopy. **(B)** WT IHGE cells or those expressing HA-inserted CXADR were exposed to Σneonicotinoids for three days. The cells were then fixed, stained with DAPI (cyan) or rabbit monoclonal anti-HA (gray: Alexa Fluor 555), and analyzed using confocal microscopy. Scale bars, 10 μm.

**Figure S4. Immunoblotting results.**
