# Supplementary material for: Neonicotinoid pesticides disrupt gingival epithelial barrier function
Source: Toxicol Rep. 2026 Mar 16;16:102238. doi: 10.1016/j.toxrep.2026.102238 (PMC13015243; doi:10.1016/j.toxrep.2026.102238)

Supplementary Table 1

Material	Source	Cat. No.
Mouse monoclonal anti-JAM1	Sigma-Aldrich	SAB4200468
Rabbit monoclonal anti-CXADR	Sino Biological	10799-R271
Mouse monoclonal anti-β-ACTIN	Sigma-Aldrich	M177-3
Mouse monoclonal anti-LAMP1	Santa Cruz	sc-20011
Alexa Fluor 488-conjugated goat anti-mouse IgG	Abcam	ab150113
Alexa Fluor 555-conjugated goat anti-mouse IgG	Invitrogen	ab150114
Alexa Fluor 555-conjugated goat anti-rabbit IgG	Invitrogen	ab150078
Alexa Fluor 633 phalloidin	Thermo Fisher Scientific	A22284
Goat anti-mouse antibody conjugated to horseradish peroxidase	Cell Signaling Technology	7076
Goat anti-rabbit antibody conjugated to horseradish peroxidase	Cell Signaling Technology	7074
pCMV-HA-inserted CXADR	Takeuchi *et al*., 2021	-
pIRES-Puro-HA-inserted CXADR	Takeuchi *et al*., 2021	-
Bafilomycin A1	Sigma-Aldrich	B1793
Fluorescein Labeling Kit-NH2	Dojindo	LK-01
FITC-*P. gingivalis* LPS	Takeuchi *et al*., 2019	-
FITC-*S. aureus* PGN	Takeuchi *et al*., 2019	-
KOD plus Neo	Toyobo	KOD-401
T4 DNA ligase	New England Biolabs	M0202

Supplementary Table 2

Primers	Sequences (5' to 3')	Anealing temperature, time (cycles)
β-ACTIN forward	GCATGGGTCAGAAGGATTCCT	58℃, 15 seconds (45)
β-ACTIN reverse	TCGTCCCAGTTGGTGACGAT	58℃, 15 seconds (45)
CXADR forward	CAGTGCCTGTTGCGTCTAAA	58℃, 15 seconds (45)
CXADR reverse	CTTTGGAGGTGGCACATCTT	58℃, 15 seconds (45)

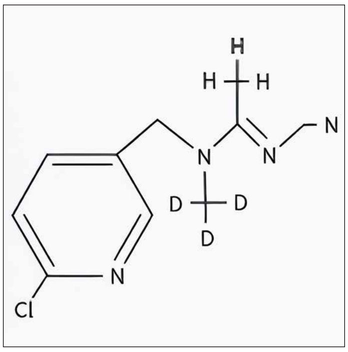

ATP-d3

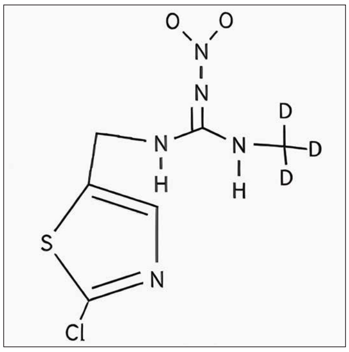

CTN-d3

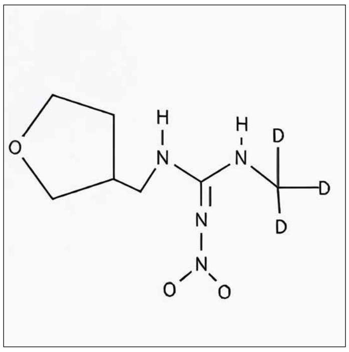

DTR-d3

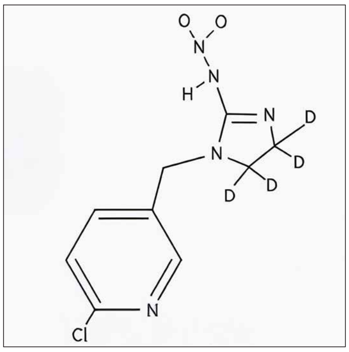

ICP-d4

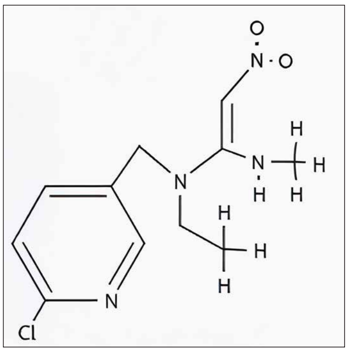

NTP

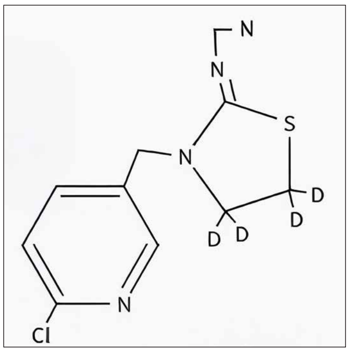

TCP-d4

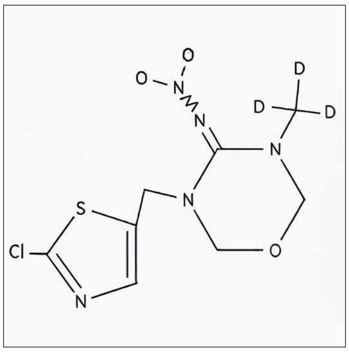

TMX-d3

Supplementary Figure 1

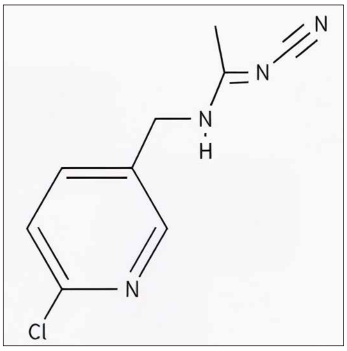

ATP

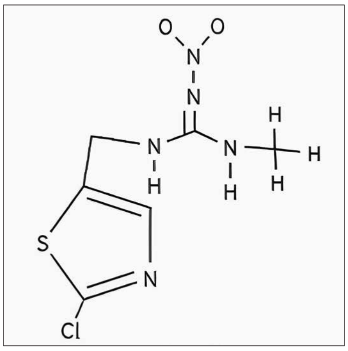

CTN

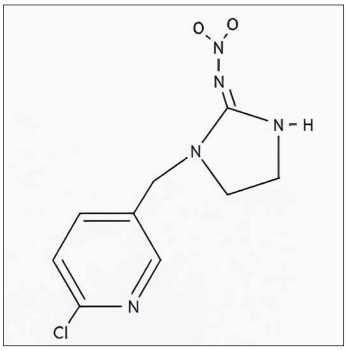

ICP

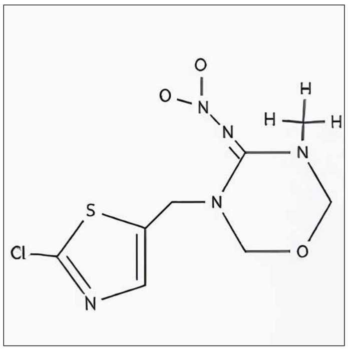

TMX

Supplementary Figure 2

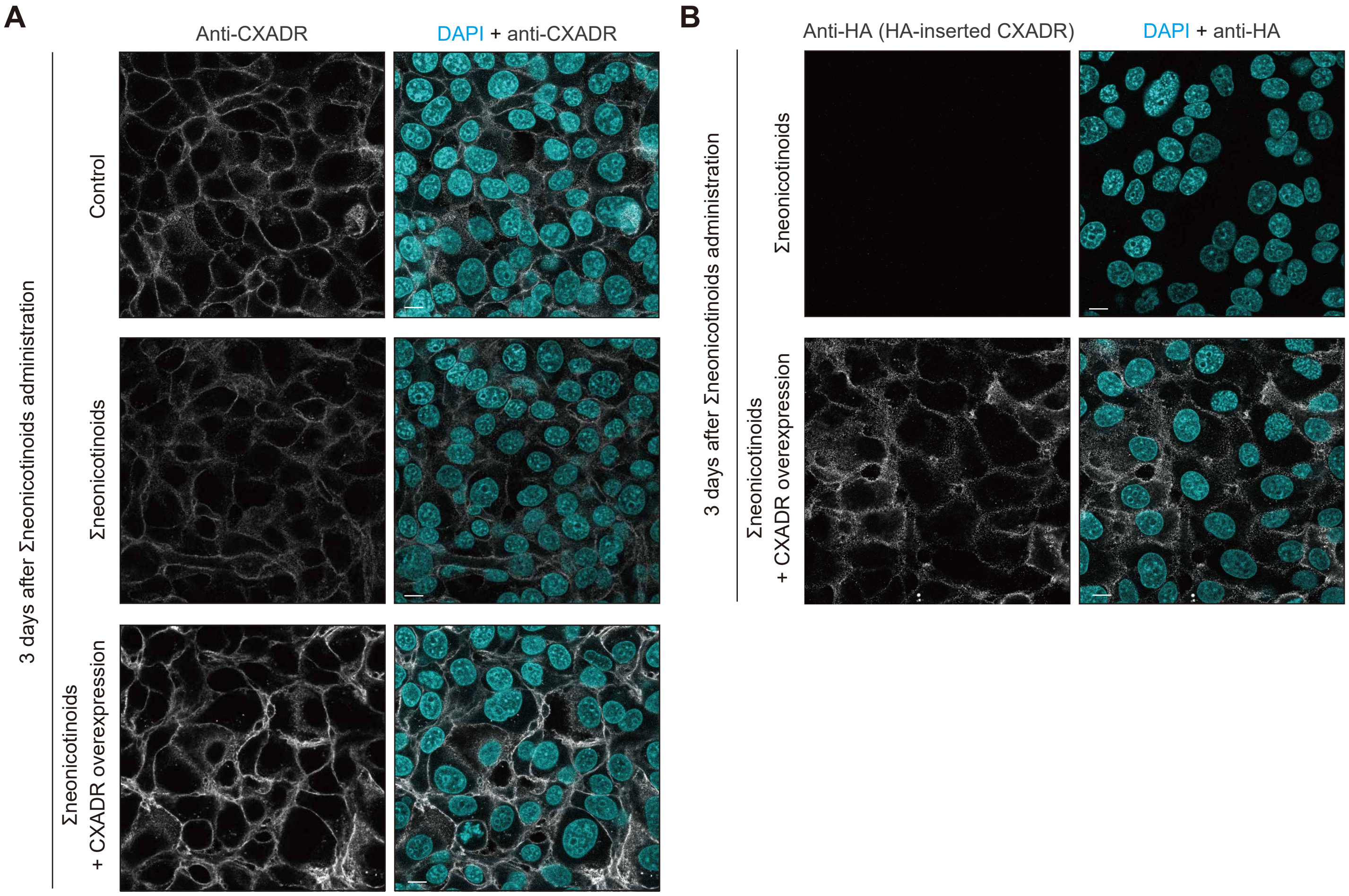

Supplementary Figure 3

Supplementary Figure 4. Immunoblotting performed in this study.

Figure 2B

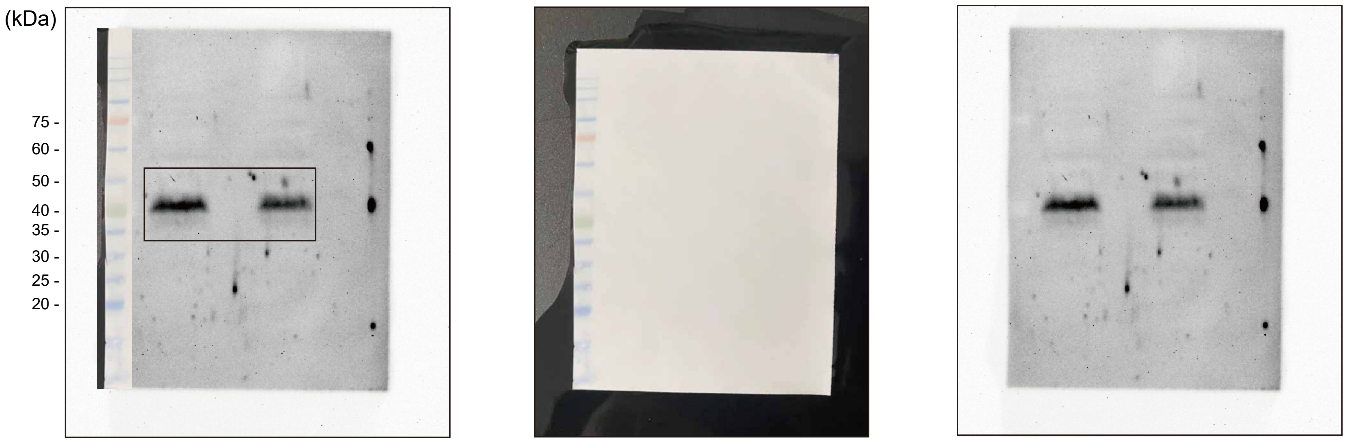

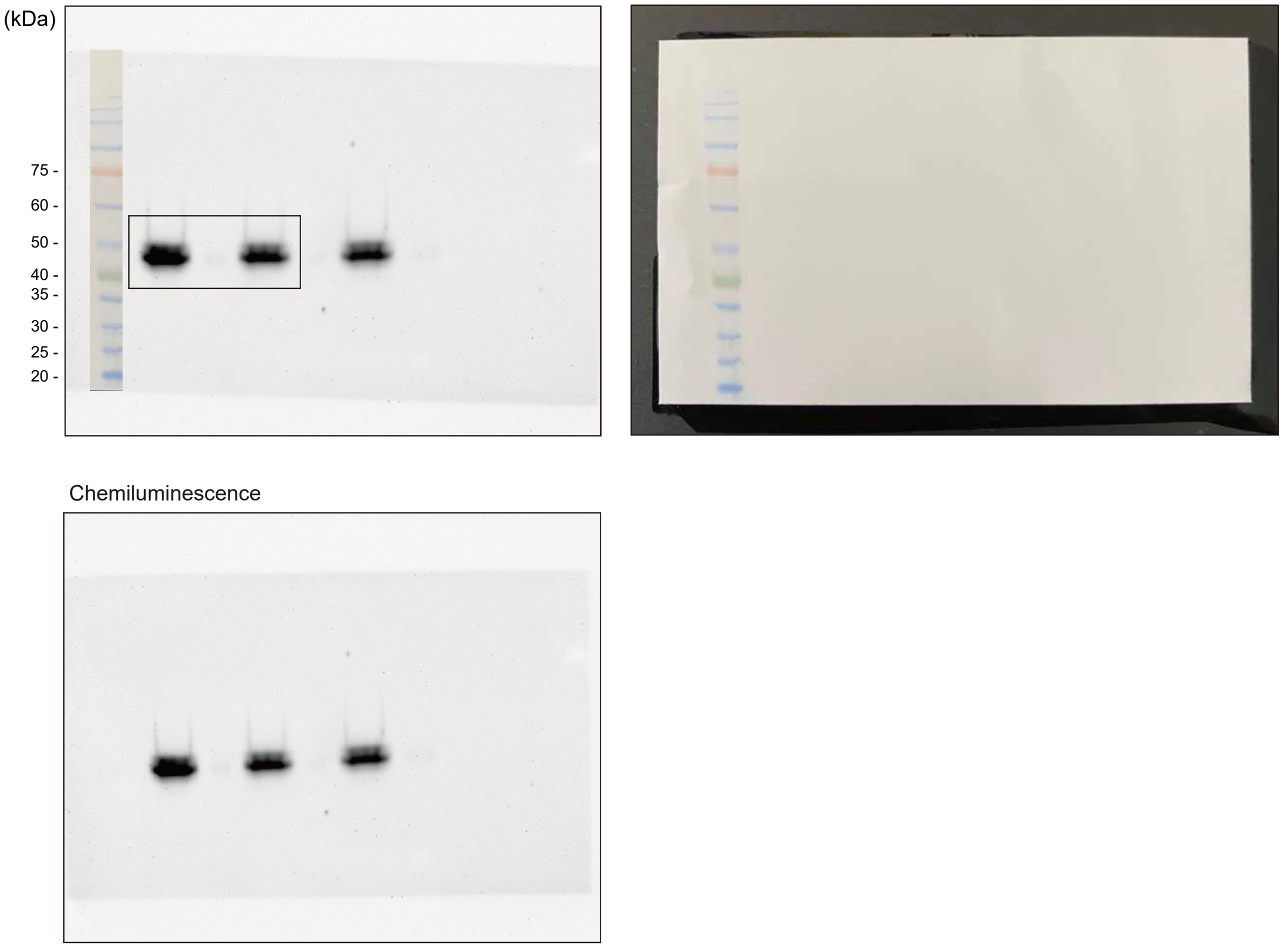

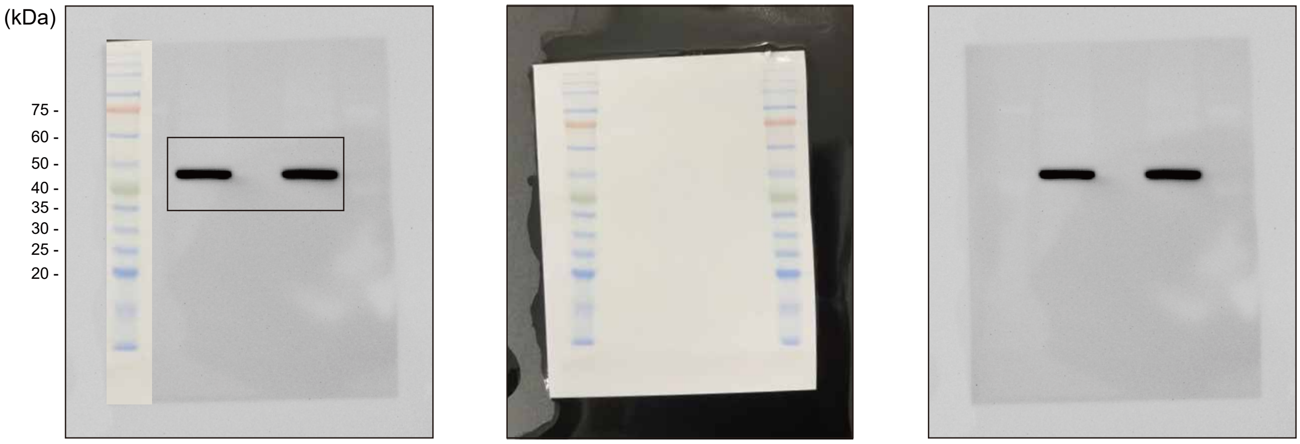

Supplement: Supplementary file 2 — Supplementary material [file mmc2.pdf]
